# Factors affecting mortality during the waiting time for kidney transplantation: A nationwide population-based cohort study using the Korean Network for Organ Sharing (KONOS) database

**DOI:** 10.1371/journal.pone.0212748

**Published:** 2019-04-12

**Authors:** Sunhwa Lee, Kyung Don Yoo, Jung Nam An, Yun Kyu Oh, Chun Soo Lim, Yon Su Kim, Jung Pyo Lee

**Affiliations:** 1 Department of Internal Medicine, Seoul National University Hospital, Seoul, Republic of Korea; 2 Department of Internal Medicine, Dongguk University College of Medicine, Gyeongju, Republic of Korea; 3 Department of Internal Medicine, Seoul National University Boramae Medical Center, Seoul, Republic of Korea; 4 Department of Critical Care Medicine, Seoul National University Boramae Medical Center, Seoul, Republic of Korea; 5 Department of Internal Medicine, Seoul National University College of Medicine, Seoul, Republic of Korea; Kaohsiung Medical University Hospital, TAIWAN

## Abstract

**Background:**

Long waiting time for deceased donor kidney transplant is inevitable due to the scarcity of donor, resulting in highlighting the importance of waiting time care. We analyzed the Korean Network for Organ Sharing (KONOS) database to assess the impact of waiting time on post-transplant survival outcomes and investigate risk factors for mortality by waiting time based on a complete enumeration survey in Korea.

**Methods:**

We analyzed all persons aged over 18 years in deceased donor kidney transplant cases enrolled in the Korean Network for Organ Sharing (KONOS) database from January 2000 to January 2015. The primary end point was all-cause mortality after enrollment.

**Results:**

Of the 24,296 wait-listed subjects on dialysis, 5,255 patients received kidney transplants from deceased donors, with a median waiting time of 4.5 years. Longer waiting times had distinct deleterious effects on overall survival after transplantation. While waiting for a transplant, patients with diabetes were more likely to die before transplantation (HR 1.515, 95% CI 1.388–1.653, p<0.001). Age was another significant risk factor for mortality. Only 56% of people aged 65 years survived after 10 years of waiting, whereas 86% of people aged 35 years survived after 10 years. Moreover, women on the waiting list were more likely to live longer than men on the list.

**Conclusions:**

More attention should be focused on patients with a higher risk of mortality while waiting for a deceased donor kidney transplant, such as patients with diabetes, those of advanced age, and those who are male.

## Introduction

The number of patients with end-stage renal disease (ESRD) is increasing rapidly at a rate of 5–7% per year [1]. In South Korea, the total number of patients receiving renal replacement therapy (RRT) has increased from 746 to 1,464 patients per million population during the past 10 years (from 2006 to 2016), whereas kidney transplantation cases have increased from 550 to 1,112 patients per million population. Although kidney transplantation is the treatment of choice due to its superior outcomes in terms of survival rate [2–4], quality of life and cost utility [5–7], the discrepancy between the supply and demand of kidneys continues to grow. As of December 2017, 19,807 patients were on the Korean Network for Organ Sharing (KONOS) waiting list for kidney transplantation. During the last decade in Korea, a 22.4% annual increase in the kidney transplantation waiting list was recorded. Consequently, the waiting time for kidney transplantation is an issue of growing importance.

In South Korea, if a patient with ESRD who should undergo renal replacement therapy prefers to undergo deceased donor kidney transplantation, each authorized clinic center evaluates the patient for recent malignancy, mental illness, drug addiction, severe liver disease, severe lung disease, active and untreated current opportunistic infection, or active ischemic heart disease [8]. If there are no contraindications for registration, they can be registered to the government-affiliated transplant waiting list (K-net, organ donation information system) managed by the Korea Centers for Disease Control and Prevention (KCDC) KONOS. After enrollment in the waiting list, the patients’ priority score is provided according to their waiting time, age (higher score for the those aged≤18 years), leukocyte antigen and ABO blood type match, history of kidney transplantation, experience of living donation, and any prior kidney transplant refusal without evident medical reasons (S1 Table). Recipients who have identical ABO blood type or compatible blood type for transfusion obtain additional scores. Patients with higher scores preferentially obtain a chance for transplantation. In contrast, there are no detailed criteria for determining candidates who receive expanded criteria donor kidneys, which satisfy one or more of the following conditions: age ≥ 60 years, GFR ≤ 60 mL/min/1.73m^2^ or serum creatinine level ≥ 3.0 mg/dL, hypotensive episode ≥ 3, proteinuria (++) ≥ 2, and non-heart beating donor [9]. The waiting time of candidates is determined according to the above allocation system in South Korea.

Longer waiting times on dialysis can negatively affect overall mortality and graft outcomes [10, 11]. Accordingly, preemptive transplantation is preferred in this context [12]. However, although living donor kidney transplantation is preferred over deceased donor kidney transplantation due to superior graft and survival outcomes [13], deceased donor kidney transplant recipients with an ESRD time of less than 6 months showed equivalent survival rates compared to those of living donor kidney transplant recipients who spent 2 years on the waiting list [14]. In other words, minimizing the waiting time for kidney transplantation is critical. Achieving this goal, however, is challenging due to a shortage of donor organs. If kidney transplant recipient candidates must wait for donor organs, then understanding the risk factors for adverse events and controlling them during that waiting period are essential.

Patients who are on the waiting list for deceased donor kidney transplantation are generally younger and healthier than patients who are not on the list [4, 15]. Therefore, the survival of potential transplant recipient patients who remain on dialysis is better than that of patients on dialysis overall. In the 2016 United States Renal Data System (USRDS) report, the most recent 2014 mortality record showed that patients on dialysis overall showed an annual mortality rate of 135.6 per 1,000 dialysis patient years, whereas patients who remain on dialysis while on the kidney transplant waiting list had a mortality rate of 53.7 per 1,000 dialysis patient years [16]. Because patients on the waiting list have different characteristics than those of patients on dialysis overall, understanding their unique characteristics and risk factors is essential. In this study, based on a complete enumeration survey in Korea, we investigated risk factors for mortality by waiting time. Moreover, the transplant probability according to various clinical factors was also explored.

## Materials and methods

### Study participants

The KONOS is a government-affiliated organization under the Centers for Disease Control (CDC) that manages deceased donor organ transplantation exclusively in South Korea. The study sample consisted of patients with ESRD who were enrolled on waiting lists for deceased or living donor kidney transplantation in 27 medical centers around the country since January 2000. Among these incident cohort patients, we included adult patients older than 18 years of age who were active on the waiting list or had already undergone deceased donor kidney transplantation. All patients were monitored from the time of registration until death or until the study end date of January 2015. Patients who became medically unsuitable for transplantation or refused a transplant remained on the waiting list until death. Patient demise was obtained from national statistics collected from information recorded at death registration.

At the time of registration, demographic and clinical data were entered into the KONOS database, including age, gender, height, body weight, start date of dialysis, cause of ESRD, ABO and Rh blood type, human leukocyte antigen (HLA) type (A, B, or DR), panel reactive antibody (PRA) positivity, and any prior kidney transplant experience.

The original data files from KONOS database are not available for public use because of the personally identifiable information. Concerning privacy risks, the data is managed only by authorized executive supervisor. The authors could perform this study after research agreement from KONOS. The raw data was provided after de-identification so that informed consent from each patients were not required. All analysis performance has never included any identifying process of patients’ personal information.

### Statistical analysis

Normally distributed continuous variables were expressed as the mean ± standard deviation (SD) and were compared using Student’s t-test. For non-normally distributed continuous variables, the median and inter-quartile range were used to express the center of the distribution, and Mann-Whitney tests were applied to compare two groups. Chi-square tests were performed to compare percentages.

For risk analysis during the waiting time, the primary outcome of our study was patient death. Comparisons between the survival curves of wait-listed patients and transplant recipients were performed using the log rank test. Moreover, we adopted time-dependent Cox regression analysis to eliminate a time-to-treatment bias when analyzing the overall mortality risk of deceased donor kidney transplant recipients according to waiting time. No patients were lost to follow-up for the primary outcome of the study, resulting in no censored cases. To analyze the risk factors during the waiting time for kidney transplant, a multivariate Cox proportional hazards model was used. The Cox proportional hazard models were adjusted for age, gender, blood type, causes of ESRD, and previous kidney transplant experience.

The probability of kidney transplantation among wait-listed patients was also analyzed. Multivariate Cox regression was adopted to examine the effects of various clinical factors on transplant probability. The primary outcome was the transplant rate, and time was censored at the transplantation date.

The statistical analysis was performed using STATA version 11.0 (StataCorp LP, College Station, TX, USA) and SPSS 18 (SPSS, Chicago, IL), and statistical significance was defined as a p value less than 0.05.

## Results

### Patient characteristics

Of the 24,296 wait-listed dialysis subjects, 5,255 patients underwent deceased donor kidney transplantation, and 19,041 patients remained on dialysis. Table 1 shows the baseline characteristics of the enrolled patients according to transplant status.

**Table 1:** Baseline characteristics of deceased donor kidney transplant recipients and characteristics of those who remained on dialysis.

Variables	Total (N = 24,296)	Recipients (n = 5,255)	Dialysis (n = 19,041)	p value
Age	48.46 ± 0.23	46.36 ± 10.84	51.97 ± 11.32	<0.001
Gender (Men)	11485 (59.6)	3048 (58.0)	11437 (60.1)	0.007
Cause of ESRD				<0.001
Diabetes	4875 (20.1)	799 (15.2)	4076 (21.4)	
Hypertensive	2709 (11.2)	782 (14.9)	1927 (10.1)	
Chronic GN	3510 (14.5)	981 (18.7)	2529 (13.3)	
PKD	406 (1.7)	47 (0.9)	359 (1.9)	
Others	4038 (16.6)	1142 (21.7)	2896 (15.2)	
Unknown	7065 (29.1)	1503 (28.6)	5562 (29.2)	
Missing data	1686 (6.9)	0 (0)	1686 (8.9)	
ABO blood type				<0.001
O	6864 (28.3)	1185(22.5)	5679(29.8)	
A	8100 (33.3)	1846(35.1)	6254(32.8)	
B	6639 (27.3)	1455(27.7)	5184(27.2)	
AB	2693 (11.1)	769(14.6)	1924(10.1)	
Rh- blood type	90 (0.4)	12(0.2)	78(0.4)	0.060
PRA positivity				
HLA Class I (-/+/missing)	-	1381/451/3423	-	
HLA Class II (-/+/missing)	-	888/321/4046	-	
BMI	-	23.43 ± 19.02	-	
Prior kidney transplant experience	1574 (6.5)	463 (29.4)	1111 (70.6)	0.419

Abbreviations: ESRD, end-stage renal disease; GN, glomerulonephritis; PKD, polycystic kidney disease; PRA, panel reactive antibody; HLA, human leukocyte antigen; BMI, body mass index.

Regarding patient demographics, kidney transplant recipients were younger than the patients who remained on dialysis (46.36 ± 10.84 vs 51.97 ± 11.32 years, p<0.001). The probability of undergoing deceased donor kidney transplantation after a 10-year wait differed according to age quartile: 1^st^ quartile (35.2 ± 5.7 years) 75.2%, 2^nd^ quartile (47.2 ± 2.6 years) 56.1%, 3^rd^ quartile (54.9 ± 2.0 years) 43.9%, and 4^th^ quartile (64.6 ± 4.8 years) 30.9%. On the other hand, the gender ratio did not show a significant difference between the groups.

The causes of ESRD in the two groups were as follows: chronic glomerulonephritis (GN) (18.7%), diabetes (15.2%), and hypertension (14.9%) for the transplant recipients, and diabetes (21.4%), chronic GN (18.7%), and hypertension (10.1%) for the remaining patients.

The proportions of ABO blood types in the two groups were also different: type A (35.1%), type B (27.7%), type O (22.5%), and type AB (14.6%) in the transplant recipients, and type A (32.8%), type O (29.8%), type B (27.2%), and type AB (10.1%) in the remaining patients. Although 28% of the patients on the overall waiting list had blood type O, these patients accounted only for 22.5% of the deceased donor kidney recipients. Compared with patients with blood type O, the probability of undergoing deceased donor kidney transplantation was 1.49-times higher for patients with blood type A, 1.41-times higher for patients with blood type B, and 2.08-times higher for patients with blood type AB. In other words, patients with blood type O must wait much longer to receive deceased donor kidney transplantation compared to patients with other blood types.

PRA positivity and body mass index (BMI) were available only in kidney transplant recipients. Only approximately a quarter (25.3%) of the recipients had information for PRA positivity.

### Overall survival in patients with deceased donor kidney transplants and in those who remained on dialysis

**Fig 1:**
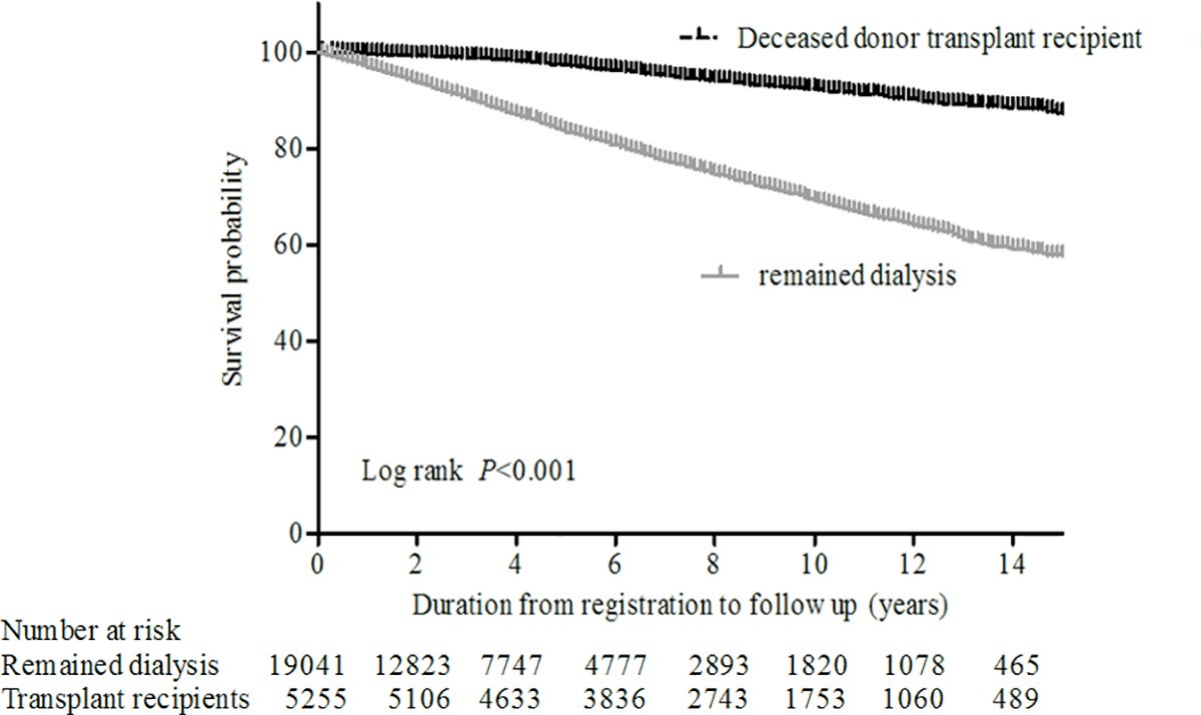
Kaplan-Meier plots comparing overall survival in patients with deceased donor kidney transplants with those who remained dialysis. The survival curves were compared using the log rank test (p<0.001).

The median waiting time for kidney transplantation from a deceased donor was 4.5 ± 2.7 years. Ten-year overall survival was 81.3% in deceased donor kidney transplant recipients and 68.1% in patients who remained on dialysis (log rank p<0.001) (Fig 1). When we evaluated the impact of waiting time for deceased donor kidney transplantation on overall mortality, a longer waiting time increased the overall mortality risk. Time-dependent Cox regression with waiting time showed that subjects in the 4^th^ quartile of waiting time (8.13 ± 1.53 years) presented a hazard ratio (HR) of 2.934 with a 95% confidence interval (CI) ranging from 1.808 to 4.763, with a p value lower than 0.001 in a comparison to subjects in the 1^st^ quartile (1.22 ± 0.71 years) (Table 2).

**Table 2:** Time-dependent cox proportional model for the overall mortality risk of deceased donor kidney transplantation.

Variable	Demographics	Hazard ratio	95% confidence interval	*P* value
Recipient Age (per a year)	46.4 ± 10.8	1.061	1.049–1.073	<0.001
Donor Age	43.4 ± 14.6	1.000	0.992–1.007	0.895
Gender (Female)	2202 (42%)	0.975	0.783–1.214	0.823
Waiting time (years)				<0.001
1st quartile (0~2.4)	1.22 ± 0.71	reference		
2nd quartile (2.4~4.5)	3.53 ± 0.57	1.935	1.349–2.768	<0.001
3rd quartile (4.5~6.4)	5.42 ± 0.56	2.502	1.649–3.796	<0.001
4th quartile (6.4~)	8.13 ± 1.53	2.934	1.808–4.763	<0.001
Prior kidney transplant experience	1574 (6.5)	1.304	0.908–1.873	0.150
Rh blood type, negative	12 (0.2%)	2.952	0.943–9.235	0.063
ABO blood type				0.655
O type	1183 (22.5%)	reference		
A type	1846 (35.1%)	1.048	0.784–1.399	0.752
B type	1455 (27.7%)	0.878	0.640–1.203	0.417
AB type	768 (14.6%)	0.995	0.694–1.428	0.980
Cause of ESRD				0.334
Diabetes	799 (15.2%)	reference		
Hypertensive	782 (14.9%)	0.799	0.559–1.141	0.216
Chronic GN	980 (18.7%)	0.723	0.500–1.044	0.083
PKD	47 (0.9%)	<0.001	0.000–0.000	0.930
Others	1142 (21.7%)	0.713	0.509–0.999	0.049
Unknown	1502 (28.6%)	0.699	0.503–0.970	0.032

Abbreviations: ESRD, end stage renal disease; GN, glomerulonephritis; PKD, polycystic kidney disease

After adjusting for recipient age, gender, waiting time, blood type, prior kidney transplant status, and cause of ESRD, we determined that older age and longer waiting time adversely influenced mortality outcomes.

### Risk factors during waiting time

During waiting time, various demographic and clinical factors were associated with increased mortality. Table 3 shows the results of univariate and multivariate Cox regression analyses of the overall mortality risk during waiting time. The statistically significant variables were old age, male gender, Rh-negative blood type, and diabetic nephropathy as the cause of ESRD. After adjusting for these variables, we found that patients with diabetes were more likely to die before transplantation (HR 1.65, 95% CI 1.51–1.80, p<0.001). Moreover, the 4^th^ quartile group of a mean age of 64.5 years had a 3.5-times higher mortality ratio than that of the 1^st^ quartile group of age 35.2 years; in other words, aging was another significant risk factor for mortality during waiting time. Female gender groups were less likely to die during waiting time than male groups. ABO Blood type and prior kidney transplant experience did not have a significant impact on overall mortality during waiting time.

**Table 3:** Univariate and multivariate regression analyses of death during waiting time before transplantation on the waiting list.

Prognostic variables	Univariate analysis			Multivariate analysis		
	Hazard ratio	P value	95% CI	Hazard ratio	P value	95% CI
Age		<0.001			<0.001	
Q1 (35.22 ± 5.70)	reference			reference		
Q2 (47.16 ± 2.55)	1.383	<0.001	1.188–1.610	1.313	<0.001	1.138–1.516
Q3 (54.94 ± 1.97)	2.305	<0.001	2.005–2.651	2.026	<0.001	1.774–2.313
Q4 (64.56 ± 4.83)	4.487	<0.001	3.946–5.104	3.523	<0.001	3.117–3.982
Sex (female)	0.779	<0.001	0.722–0.840	0.803	<0.001	0.744–0.866
ABO blood type		0.762			0.858	
O	reference			reference		
A	0.987	0.781	0.900–1.083	1.031	0.508	0.942–1.129
B	1.038	0.446	0.943–1.142	1.019	0.690	0.927–1.121
AB	1.012	0.865	0.886–1.156	1.052	0.439	0.925–1.198
Rh blood type (Rh-)	1.638	0.028	1.055–2.543	1.608	0.027	1.057–2.448
ESRD cause		<0.001			<0.001	
DM	reference			reference		
Hypertension	0.369	<0.001	0.314–0.434	0.460	<0.001	0.395–0.535
chronic GN	0.315	<0.001	0.272–0.365	0.410	<0.001	0.356–0.472
PKD	0.455	<0.001	0.297–0.696	0.368	<0.001	0.221–0.614
Others	0.519	<0.001	0.462–0.584	0.599	<0.001	0.534–0.672
Unknown	0.647	<0.001	0.585–0.715	0.705	<0.001	0.638–0.779
Prior kidney transplant experience	0.954	0.432	0.849–1.073	1.054	0.377	0.938–1.186

Abbreviations: Q1, 1st quartile; Q2, 2nd quartile; Q3, 3rd quartile; Q4, 4th quartile; ESRD, end-stage renal disease; DM, diabetic mellitus; GN, glomerulonephritis; PKD, polycystic kidney disease.

### Probability of undergoing transplantation

**Fig 2:**
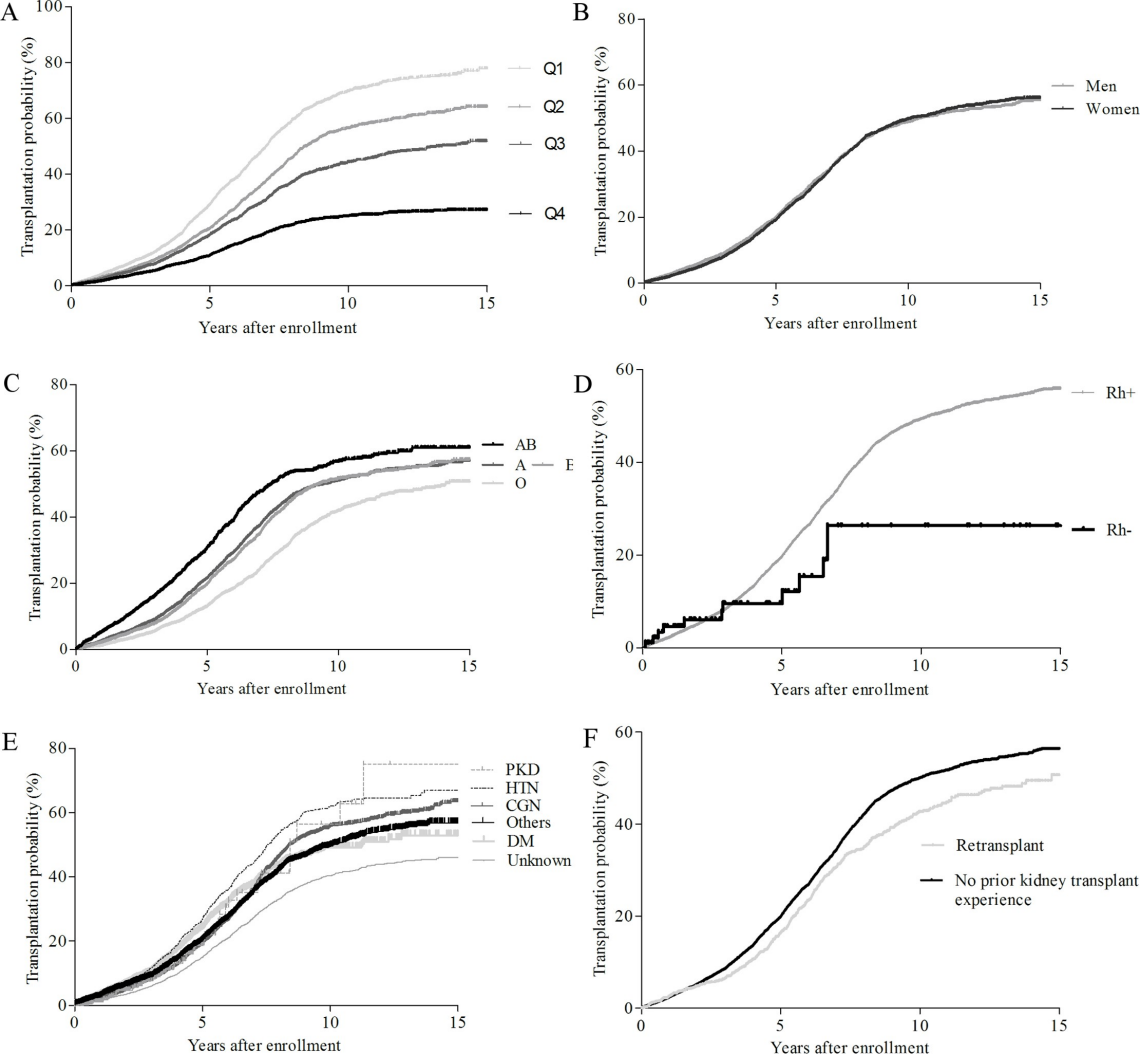
**Cumulative probability to undergo deceased donor kidney transplantation on the waiting list according to age quartile (A), gender (B), ABO blood type (C), Rh blood type (D), cause of ESRD (E), and prior kidney transplant experience (F).** Abbreviations: Q1, first quartile, age of 18 to 42; Q2, second quartile, age of 43 to 51; Q3, third quartile, age of 52 to 58; Q4, fourth quartile, age of 59 to 96; PKD, polycystic kidney disease; HTN, hypertension; CGN, chronic glomerulonephritis; DM, diabetic mellitus.

We performed multivariate regression analysis on the success rate of receiving a kidney transplant according to various demographic variables to analyze which patients received deceased donor kidney allografts earlier (Table 4). Our analysis showed that patients who were of advanced age, had diabetes, had blood type O, or had undergone previous transplantation tended to receive deceased donor kidneys later (Fig 2).

**Table 4:** Multivariate regression analysis of the probability of receiving a deceased donor kidney on the waiting list.

Prognostic variables	Hazard ratio (95% CI)	p value
Age		<0.001
Q1 (35.22 ± 5.70)	reference	
Q2 (47.16 ± 2.55)	0.831 (0.775–0.891)	<0.001
Q3 (54.94 ± 1.97)	0.704 (0.653–0.760)	<0.001
Q4 (64.56 ± 4.83)	0.397 (0.363–0.435)	<0.001
ABO blood type		<0.001
O	reference	
A	1.368 (1.270–1.475)	<0.001
B	1.311 (1.212–1.418)	<0.001
AB	1.698 (1.545–1.866)	<0.001
ESRD cause		<0.001
DM	reference	
Hypertension	1.110 (1.005–1.226)	0.039
chronic GN	0.718 (0.652–0.790)	<0.001
PKD	0.869 (0.647–1.166)	0.349
Others	0.678 (0.618–0.744)	<0.001
Unknown	0.669 (0.613–0.730)	<0.001
Prior kidney transplant experience	0.755 (0.686–0.831)	<0.001

Abbreviations: Q1, 1^st^ quartile; Q2, 2^nd^ quartile; Q3, 3^rd^ quartile; Q4, 4^th^ quartile; ESRD, end-stage renal disease; DM, diabetes mellitus; GN, glomerulonephritis; PKD, polycystic kidney disease.

## Discussion

In the present study, we performed risk factor analysis based on waiting time for 24,296 individuals with ESRD on the waiting list for renal transplantation. Longer waiting periods had an obvious negative effect on overall mortality after transplantation. During that time, patients who were male, of advanced age, or had diabetes were vulnerable to death. We also found that patients who were of advanced age, had diabetes, had blood type O, or had prior kidney transplant experience received donor kidneys later than others on the list.

Younger and healthier patients tend to receive kidney transplantation earlier, and longer waiting times should therefore be associated with higher mortality rates. However, in our analysis, we adjusted for demographic and clinical factors, such as age and the cause of ESRD. As a result, longer waiting time was identified as an independent risk factor for post-transplant mortality, which has also been shown in previous studies [10, 11].

This study mainly showed which risk factors may affect mortality or kidney transplantation rates during waiting times. The prevalence of mortality is known to increase significantly with age or diabetes in patients with ESRD [17–19]. Although patients with diabetes account for 48.4% of new patients with ESRD in Korea [18], the proportion of these patients on the waiting list is 20.1%. Because of the high morbidity of these patients with diabetes, they may have a reduced likelihood of being enrolled on the waiting list. Although relatively healthy patients with diabetes are on the registry, they show a higher mortality rate during the waiting time than do patients with any other ESRD causes. The Korean Society of Nephrology (KSN) registry data also showed that the 5-year survival rate of patients with diabetes and ESRD was 53%, whereas other patients with ESRD and chronic glomerulonephritis or hypertensive nephrosclerosis had survival rates of 78% and 69.8%, respectively [20]. On our KONOS waiting list, the 5-year survival rate during the waiting time was 77% for patients with diabetes, 91% for patients with hypertensive nephrosclerosis, and 93% for patients with glomerulonephritis.

In general, women have a survival advantage over men in the general population. Even in patients with chronic kidney disease, women tend to progress to ESRD at a slower rate than men do, regardless of disease etiology [21]. However, in the dialysis population, women lost this benefit [22] and showed survival rates similar to those in men. Even if kidney transplantation is performed, this survival benefit cannot be recovered in women [23]. Furthermore, young women with ESRD (under age 35 years) tend to have higher overall or non-cardiovascular mortality rates than do men on dialysis. This pattern was not different in the Korean dialysis population [24]. However, although no gender advantage was observed for the overall mortality rate after deceased donor kidney transplantation in our study, men were 1.25-times more likely to die than women are during waiting time. This result may have been affected by influences other than clinical factors. Some studies have shown that the statistical significance of mortality rates during waiting times may be influenced by insurance status, income, family support, and socioeconomic status [25, 26]. Unfortunately, this personal information was not provided by the KONOS to protect personal identification information. Although the reason why men are more likely to die during waiting time than are women is unclear (unlike the survival benefit of women in the general dialysis population), we propose that socioeconomic conditions may contribute to this difference.

In our study, we determined that patients with blood type O or prior kidney transplant experience were disadvantaged in terms of receiving a deceased donor kidney in a timely manner. Patients with advanced age or diabetes may acquire more comorbidities as waiting time progresses, so a reduced probability of undergoing transplantation over time is expected. However, patients with blood type O suffer from a disadvantage in the prioritization of receiving deceased donor kidney allografts due to biological differences. ABO-compatible kidney transplantation rules allocate blood type O kidneys to non-blood type O recipients; consequently, patients with blood type O on dialysis who can only receive kidneys from the same blood type have a lesser likelihood of receiving these organs. According to the annual statistical report by the KONOS, 4~7% of deceased donor kidney transplantations were performed on non-identical–but ABO-compatible–blood types in the last 5 years [27]. As a result, wait-listed patients with blood type O wait longer to receive deceased donor kidneys than patients with other blood types, leading to worse outcomes. A similar blood type O problem has been described in the Eurotransplant Kidney Allocation System, and some studies have reported that the current allocation system should be revised [28]. On the other hand, in the United States, the Organ Procurement and Transplantation Network already accounts for this concept in its allocation system. A restriction mandates that kidneys from donors with blood type B or O are allocated to recipients with identical blood types, except in zero-HLA mismatch category cases [29, 30]. In the United States, blood type B candidates have the longest waiting time (6 years in 2014) compared to those with other blood types (type A, 3 years; type AB, 2 years; type O, 5 years) [31], while in South Korea, blood type O candidates have the longest waiting time (type O, 5.2 years; type A, 4.4 years; type B 4.6 years; type AB, 3.4 years; in 2014). Because the United States is under special condition wherein a majority of deceased kidney donors are white, of whom only 9% of them have blood type B, there exist disparities for blood type B candidates in access to transplantation [32]. Therefore, to protect blood group B candidates, of whom a majority are ethnic minorities, the national kidney allocation system assigns A2/A2B kidney to B recipients preferentially. Although it comprises a minor portion, A2/A2B →B deceased donor kidney transplantations increased from 14 (1.95%) to 53 (8.93%) among blood group B deceased donor kidney transplantation [33]. In South Korea, the fairness of our organ allocation system warrants re-evaluation to avoid reverse discrimination based on biological differences.

In addition, not only patients with blood type O or history of kidney transplantation were less likely to undergo deceased donor kidney transplant in a timely manner but also those with causes of ESRD other than DM tended to undergo deceased kidney at a later time. Table 4 shows that candidates with hypertensive and diabetic ESRD are ranked first and second for transplantation probability during waiting time, whereas chronic glomerulonephritis or other causes were ranked next, although the Korean kidney allocation system does not include causes of ESRD as rating criteria. If we look into the proportion of ABO blood type in each cause of ESRD, diabetic patients have lesser portion of blood type O population compared to non-diabetic ESRD patients (S2 Table). Recent research reported that people with blood type O have a lower risk of developing type 2 diabetes mellitus [34]. Because, blood type O candidates tend to wait longer to receive a deceased kidney, any group composed of a larger proportion of blood type O candidates will likely to wait longer. Therefore, this is the possible reason patients with causes of ESRD other than diabetes are less likely to undergo transplantation.

In our study, the analysis was limited in at least three respects. First, missing or unknown data represented 36% of the data on ESRD causes. Another limitation was knowledge regarding the origin of kidney disease in all patients with ESRD because kidney biopsy was not performed in all patients to determine the cause of ESRD. Nevertheless, the fact that more than one-third of the data were unknown or missing causes an interpretation bias. Second, socioeconomic status was not provided for the wait-listed candidates. In Korea, at least 10 thousand dollars per patient is required for kidney transplantation surgery, even if these patients benefit from national health services [35]. Therefore, barriers to entry on the transplant waiting list exist for economically disadvantaged individuals. Moreover, family support or insurance status may affect mortality during waiting time or may affect transplantation rates; however, such parameters were not included in this analysis. Third, comorbidities such as diabetes and ischemic heart disease were not adjusted for in survival and transplant probability analyses. Although these are highly influential factors for the outcome, we do not have this information from the KONOS.

In conclusion, this study indicated the risk factors of mortality during waiting time for deceased donor kidney transplantation and the characteristics of patients who received deceased donor kidney transplantation after longer waiting times. Considering the deleterious effect of longer waiting time on mortality, identifying vulnerable patients during the waiting time and providing more intensive care for these patients are important.

## Supporting information

S1 File Extracted raw data of KONOS database. (ZIP)

S1 Table Deceased donor kidney allocation scoring system in South Korea. (DOCX)

S2 Table The proportion of ABO blood type of diabetic and non-diabetic ESRD patients in the waiting list in our study group. (DOCX)

## References

[1] U.S. Renal Data System, USRDS 2010 Annual Data Report: Atlas of Chronic Kidney Disease and End-Stage Renal Disease in the United States, National Institutes of Health, National Institute of Diabetes and Digestive and Kidney Diseases, Bethesda, MD, 2010. Available from: https://www.usrds.org/atlas10.aspx Cited 7 February 2018.

[2] Medin C, Elinder CG, Hylander B, Blom B, Wilczek H. Survival of patients who have been on a waiting list for renal transplantation. *Nephrol Dial Transplant* 2000; 15(5): 701–4. 10809814 10.1093/ndt/15.5.701

[3] Port FK, Wolfe RA, Mauger EA, Berling DP, Jiang K. Comparison of survival probabilities for dialysis patients vs cadaveric renal transplant recipients. *Jama* 1993; 270(11): 1339–43. 8360969

[4] Wolfe RA, Ashby VB, Milford EL, Ojo AO, Ettenger RE, Agodoa LY, et al Comparison of mortality in all patients on dialysis, patients on dialysis awaiting transplantation, and recipients of a first cadaveric transplant. *N Engl J Med* 1999; 341(23): 1725–30. 10.1056/NEJM199912023412303 10580071

[5] Jofre R, López-Gómez JM, Moreno F, Sanz-Guajardo D, Valderrábano F. Changes in quality of life after renal transplantation. *Am J Kidney Dis* 1998; 32(1): 93–100. 9669429 10.1053/ajkd.1998.v32.pm9669429

[6] Laupacis A, Keown P, Pus N, Krueger H, Ferguson B, Wong C, et al A study of the quality of life and cost-utility of renal transplantation. *Kidney Int* 1996; 50(1): 235–42. 8807593 10.1038/ki.1996.307

[7] Lee AJ, Morgan CL, Conway P, Currie CJ. Characterisation and comparison of health-related quality of life for patients with renal failure. *Curr Med Res Opin* 2005; 21(11): 1777–83. 10.1185/030079905X65277 16307698

[8] Kim C-D. Kidney Transplantation. Korean J Med. 2014;86(2):142–51.

[9] Organ transplant management guideline 2016: Korean Network for Organ Sharing, Ministry of Health and Welfare. Available from: https://www.konos.go.kr/konosis/common/bizlogic.jsp cited 7 February 2018.

[10] Cosio FG, Alamir A, Yim S, Pesavento TE, Falkenhain ME, Henry ML, et al Patient survival after renal transplantation: I. The impact of dialysis pre-transplant. *Kidney Int* 1998; 53(3): 767–72. 10.1046/j.1523-1755.1998.00787.x 9507225

[11] Meier-Kriesche HU, Port FK, Ojo AO, Rudich SM, Hanson JA, Cibrik DM, et al Effect of waiting time on renal transplant outcome. *Kidney Int* 2000;58(3):1311–7. 10.1046/j.1523-1755.2000.00287.x 10972695

[12] Kasiske BL, Snyder JJ, Matas AJ, Ellison MD, Gill JS, Kausz AT. Preemptive kidney transplantation: the advantage and the advantaged. *J Am Soc Nephrol* 2002; 13(5): 1358–64. 11961024 10.1097/01.asn.0000013295.11876.c9

[13] Medin C, Elinder CG, Hylander B, Blom B, Wilczek H. Survival of patients who have been on a waiting list for renal transplantation. *Nephrol Dial Transplant* 2000; 15(5): 701–4. 10809814 10.1093/ndt/15.5.701

[14] Meier-Kriesche H-U, Kaplan B. Waiting time on dialysis as the strongest modifiable risk factor for renal transplant outcomes: A Paired Donor Kidney Analysis1. *Transplantation* 2002; 74(10): 1377–81. 10.1097/01.TP.0000034632.77029.91 12451234

[15] Gaylin DS, Held PJ, Port FK, Hunsicker LG, Wolfe RA, Kahan BD, et al The impact of comorbid and sociodemographic factors on access to renal transplantation. *Jama* 1993; 269(5): 603–8. 8421364

[16] Saran R, Robinson B, Abbott KC, Agodoa LY, Albertus P, Ayanian J, et al US renal data system 2016 annual data report: epidemiology of kidney disease in the United States. *Am J Kidney Dis* 2017; 69(3): A7–A8. 28236831 10.1053/j.ajkd.2016.12.004 PMC6605045

[17] Foley RN, Parfrey PS, Sarnak MJ. Clinical epidemiology of cardiovascular disease in chronic renal disease. *Am J Kidney Dis* 1998; 32(5): S112–S9. 9820470 10.1053/ajkd.1998.v32.pm9820470

[18] Sarnak MJ, Levey AS, Schoolwerth AC, Coresh J, Culleton B, Hamm LL, et al Kidney disease as a risk factor for development of cardiovascular disease. *Circulation* 2003; 108(17): 2154–69. 10.1161/01.CIR.0000095676.90936.80 14581387

[19] Tien K-J, Lin Z-Z, Chio C-C, Wang J-J, Chu C-C, Sun Y-M, et al Epidemiology and Mortality of New-Onset Diabetes After Dialysis. *Diabetes care* 2013; 36(10): 3027–32. 10.2337/dc12-2148 23723355 PMC3781558

[20] Jin D-C, Yun S-R, Lee SW, Han S-W, Kim W, Park J. Current characteristics of dialysis therapy in Korea: 2015 registry data focusing on elderly patients. *Kidney Res Clin Pract* 2016; 35(4): 204–11. 10.1016/j.krcp.2016.09.006 27957414 PMC5142391

[21] Neugarten J, Acharya A, Silbiger SR. Effect of gender on the progression of nondiabetic renal disease a meta-analysis. *J Am Soc Nephrol* 2000; 11(2): 319–29. 10665939 10.1681/ASN.V112319

[22] Villar E, Remontet L, Labeeuw M, Ecochard R. Effect of age, gender, and diabetes on excess death in end-stage renal failure. *J Am Soc Nephrol* 2007; 18(7): 2125–34. 10.1681/ASN.2006091048 17582163

[23] Øien CM, Reisæter AV, Os I, Jardine A, Fellström B, Holdaas H. Gender‐associated risk factors for cardiac end points and total mortality after renal transplantation: post hoc analysis of the ALERT study. *Clin Transplant* 2006; 20(3): 374–82. 10.1111/j.1399-0012.2006.00496.x 16824157

[24] Choi H, Kim M, Kim H, Lee JP, Lee J, Park JT, et al Excess mortality among patients on dialysis: comparison with the general population in Korea. *Kidney Res Clin Pract* 2014; 33(2): 89–94. 10.1016/j.krcp.2014.04.001 26877956 PMC4714183

[25] Port FK, Wolfe RA, Levin NW, Guire KE, Ferguson CW. Income and survival in chronic dialysis patients. *ASAIO Trans* 1990; 36(3): M154–7. 2252648

[26] McClellan WM, Anson C, Birkeli K, Tuttle E. Functional status and quality of life: predictors of early mortality among patients entering treatment for end stage renal disease. *J Clin Epidemiol* 1991; 44(1): 83–9. 1986062 10.1016/0895-4356(91)90204-m

[27] 2015 KONOS annual report: Korea Centers for Disease Control and Prevention Web site. Available from: http://cdc.go.kr/CDC/cms/content/mobile/75/24275_view.html Cited 7 February 2018.

[28] Glander P, Budde K, Schmidt D, Fuller TF, Giessing M, Neumayer H-H, et al The ‘blood group O problem’in kidney transplantation—time to change? *Nephrol Dial Transplant* 2010; 25(6): 1998–2004. 10.1093/ndt/gfp779 20100733

[29] Israni AK, Salkowski N, Gustafson S, Snyder JJ, Friedewald JJ, Formica RN, et al New national allocation policy for deceased donor kidneys in the United States and possible effect on patient outcomes. *J Am Soc Nephrol* 2014; 25(8): 1842–8. 10.1681/ASN.2013070784 24833128 PMC4116061

[30] Organ Procurement and Transplantation Network policies. Health Resources and Services Administration, U.S. Department of Health & Human Services Web site. Available from: https://optn.transplant.hrsa.gov/governance/policies Cited 7 February 2018.

[31] Hart A, Smith JM, Skeans MA, Gustafson SK, Stewart DE, Cherikh WS, et al Kidney. American journal of transplantation: official journal of the American Society of Transplantation and the American Society of Transplant Surgeons. 2016;16 Suppl 2:11–46. 10.1111/ajt.13666 PMC5541687 26755262

[32] Nelson PW, Shield CF, Muruve NA, Murillo D, Warady BA, Aeder MI, et al Increased access to transplantation for blood group B cadaveric waiting list candidates by using A2 kidneys: time for a new national system? American Journal of Transplantation. 2002;2(1):94–9. 12095063 10.1034/j.1600-6143.2002.020115.x

[33] Martins PN, Mustian MN, MacLennan PA, Ortiz JA, Akoad M, Caicedo JC, et al Impact of the new kidney allocation system A2/A2B→ B policy on access to transplantation among minority candidates. American Journal of Transplantation. 2018. 10.1111/ajt.14719 PMC6105461 29509285

[34] Fagherazzi G, Gusto G, Clavel-Chapelon F, Balkau B, Bonnet F. ABO and Rhesus blood groups and risk of type 2 diabetes: evidence from the large E3N cohort study. Diabetologia. 2015;58(3):519–22. 10.1007/s00125-014-3472-9 25533388

[35] Kim MS. Expansion of health insurance coverage for organ transplantation. Ministry of Strategy and Finance Republic of Korea, All Public Information In-One web site. Available from: http://www.alio.go.kr/popSusiViewB1040.do Cited 7 February 2018.

