# Supplementary material for: Factors affecting mortality during the waiting time for kidney transplantation: A nationwide population-based cohort study using the Korean Network for Organ Sharing (KONOS) database
Source: PLoS One. 2019 Apr 12;14(4):e0212748. doi: 10.1371/journal.pone.0212748 (PMC6461220; doi:10.1371/journal.pone.0212748)
Supplement: S1 Table — (DOCX) [file pone.0212748.s002.docx]

**S1 Table.** Deceased donor kidney allocation scoring system in South Korea.

|  | Parameters | points |
| --- | --- | --- |
| Blood type | Identical | 10 points |
|  | Compatible for blood transfusion | 5 points |
| Leukocyte antigen | HLA-DR | 1 point per match |
|  | HLA-A or B | 0.5 point per match |
| Recipient’s age | ≤ 11 years | 4 points |
|  | 12-18 years | 3 points |
| Waiting time |  | (number of total candidates – candidate rank)/ number of total candidates x 1 point |
| Prior HLA crossmatch test | Positive results were more than twice | 2 points |
| Experience of living donation* | Oneself | 4 points |
|  | Spouse or lineal ascendant and descendant | 4 points |
|  | Siblings | 3 points |
|  | First cousins or aunt/uncle | 2 points |
| Prior kidney transplant | More than once | 2 points |
| Refusal of transplant without evident medical reasons | Per event | - 0.1 point |

Abbreviations: HLA, human leukocyte antigen

*The highest score is counted for the rating.
