# Supplementary material for: Factors affecting mortality during the waiting time for kidney transplantation: A nationwide population-based cohort study using the Korean Network for Organ Sharing (KONOS) database
Source: PLoS One. 2019 Apr 12;14(4):e0212748. doi: 10.1371/journal.pone.0212748 (PMC6461220; doi:10.1371/journal.pone.0212748)
Supplement: S2 Table — (DOCX) [file pone.0212748.s003.docx]

**S2 Table.** The proportion of ABO blood type of diabetic and non-diabetic ESRD patients in the waiting list in our study group

| Causes of ESRD | O | A | B | AB | Total | P-value (chi-square) | |
| --- | --- | --- | --- | --- | --- | --- | --- |
| Hypertension | 744 | 932 | 735 | 298 | 2709 | 0.051 |  |
|  | 27.5% | 34.4% | 27.1% | 11.0% | 100.0% |  |  |
| Diabetes | 1231 | 1638 | 1411 | 595 | 4875 | Reference |  |
|  | **25.3%** | 33.6% | 28.9% | 12.2% | 100.0% |  |  |
| GN | 1050 | 1163 | 946 | 350 | 3509 | <0.001 |  |
|  | **29.9%** | 33.1% | 27.0% | 10.0% | 100.0% |  |  |
| PKD | 114 | 141 | 108 | 43 | 406 | 0.417 |  |
|  | 28.1% | 34.7% | 26.6% | 10.6% | 100.0% |  |  |
| Others | 1149 | 1339 | 1061 | 489 | 4038 | 0.003 |  |
|  | **28.5%** | 33.2% | 26.3% | 12.1% | 100.0% |  |  |
| Total | 6862 | 8100 | 6639 | 2692 | 24293 |  | |
|  | 28.2% | 33.3% | 27.3% | 11.1% | 100.0% |  | |

Abbreviations: ESRD, end stage renal disease; GN, glomerulonephritis; PKD, polycystic kidney disease
